# Chromosomally-Encoded *Yersinia pestis* Type III Secretion Effector Proteins Promote Infection in Cells and in Mice

**DOI:** 10.3389/fcimb.2019.00023

**Published:** 2019-02-22

**Authors:** Sara Schesser Bartra, Cherish Lorica, Lianfen Qian, Xin Gong, Wael Bahnan, Henry Barreras Jr., Rosmely Hernandez, Zhongwei Li, Gregory V. Plano, Kurt Schesser

**Affiliations:** ^1^Department of Microbiology and Immunology, University of Miami Miller School of Medicine, Miami, FL, United States; ^2^Division of Pediatric Infectious Diseases, Department of Pediatrics, University of Miami Miller School of Medicine, Miami, FL, United States; ^3^Department of Mathematics, Charles E. Schmidt College of Science, Florida Atlantic University, Boca Raton, FL, United States; ^4^Department of Biomedical Science, Charles E. Schmidt College of Medicine, Florida Atlantic University, Boca Raton, FL, United States

**Keywords:** *Yersinia*, type III secretion, pathogenesis, leucine rich repeats, plague, mice

## Abstract

*Yersinia pestis*, the causative agent of plague, possesses a number of virulence mechanisms that allows it to survive and proliferate during its interaction with the host. To discover additional infection-specific *Y. pestis* factors, a transposon site hybridization (TraSH)-based genome-wide screen was employed to identify genomic regions required for its survival during cellular infection. In addition to several well-characterized infection-specific genes, this screen identified three chromosomal genes (*y3397, y3399*, and *y3400*), located in an apparent operon, that promoted successful infection. Each of these genes is predicted to encode a leucine-rich repeat family protein with or without an associated ubiquitin E3 ligase domain. These genes were designated *Yersinia* leucine-rich repeat gene A (*ylrA*), B (*ylrB*), and C (*ylrC*). Engineered strains with deletions of y3397 (*ylrC*), y3399 (*ylrB*), or y3400 (*ylrA*), exhibited infection defects both in cultured cells and in the mouse. C-terminal FLAG-tagged YlrA, YlrB, and YlrC were secreted by *Y. pestis* in the absence but not the presence of extracellular calcium and deletions of the DNA sequences encoding the predicted N-terminal type III secretion signals of YlrA, YlrB, and YlrC prevented their secretion, indicating that these proteins are substrates of the type III secretion system (T3SS). Further strengthening the connection with the T3SS, YlrB was readily translocated into HeLa cells and expression of the YlrA and YlrC proteins in yeast inhibited yeast growth, indicating that these proteins may function as anti-host T3S effector proteins.

## Introduction

*Yersinia pestis*, the etiologic agent of plague, causes a variety of serious diseases in humans and animals. The clinical syndromes in humans include bubonic, pneumonic, and septicemic plague (Perry and Fetherston, 1997). Fleas transmit *Y. pestis* from infected domestic rats to humans causing bubonic plague. *Y. pestis* also can be transmitted via respiratory secretions following contact with another infected human, leading to pneumonic plague. Currently, plague is still a public health threat in certain regions of Asia, Africa, North and South America (Centers for Disease Control Prevention., 2013). In recent years in the U.S., most cases of plague have occurred in children in whom diagnosis has been delayed. Among 183 U.S. pediatric cases from 1947 to 2001, 91% presented primarily as bubonic and one third of these cases developed secondary complications, such as sepsis, meningitis, and pneumonia. Children were more likely than adults to manifest with bubonic plague (91 vs. 79%), develop complications (32 vs. 27%), and to die (17 vs. 14%) (Dennis and Chow, 2004). Because plague is highly contagious, *Y. pestis* can be used in biological warfare and is considered a Category A agent of bioterrorism (Inglesby et al., 2000).

Among the virulence factors identified in *Y. pestis*, the plasmid pCD1 encoded type III secretion system (T3SS) is one of the best-studied. The T3SS is a complex protein machinery shared by numerous Gram-negative pathogens that injects effector proteins directly into the cytosol of an infected eukaryotic cell (Cornelis, 2002a). The translocation of effector proteins requires direct contact between the bacterium and the host cell. In *Y. pestis*, the T3S apparatus is called the Ysc injectosome and the effector proteins are termed Yops (Yersinia outer proteins). The Yops disrupt host signaling pathways that normally lead to bacterial phagocytosis and the production of pro-inflammatory cytokines (Cornelis and Van Gijsegem, 2000). To date, six Yop effectors have been identified—YopH, YopE, YopT, YpkA/YopO, YopP/YopJ, and YopM (Cornelis, 2002b). Following its identification as a virulence factor in the mouse model, YopM was initially described as an inhibitor of platelet aggregation (Leung and Straley, 1989). Later YopM was linked to a variety of activities associated with the host innate immune response including depletion of inflammatory monocytes and natural killer (NK) cells and induction of specific cytokines (Kerschen et al., 2004; Ye et al., 2009; McPhee et al., 2010). Once translocated into eukaryotic cells, YopM directly interacts with a number of host proteins including kinases and capase-1 to inhibit signaling pathways and inflammasome activation (McDonald et al., 2003; Hentschke et al., 2010; LaRock and Cookson, 2012; Chung et al., 2014).

Structurally YopM is primarily composed of a number of leucine-rich repeats (LRR) (Evdokimov et al., 2001). Recently it has been shown that *Y. pestis*, as well as a number of other *Yersinia* species (but not *Y. enterocolitica*), possesses a chromosomal locus that potentially encodes three YopM-like proteins (Hu et al., 2016). Sequence analysis showed that the plasmid-encoded YopM was evolutionarily distinct from these three chromosomal-encoded YopM-like proteins and that two of these YopM-like proteins possessed E3 ligase domains in their C-terminal regions (Hu et al., 2016). Here we describe the independent identification and characterization of the genes encoding these three chromosomally-encoded YopM-like proteins in an unbiased screen for *Y. pestis* factors that promote its survival following its infection of macrophages.

## Materials and Methods

### Bacterial Strains

The *Escherichia coli* DH5α and *Y. pestis* strains were routinely grown in heart infusion broth (HIB) or on tryptose blood agar (TBA) base plates (Difco, Detroit, MI) at 27°C (*Y. pestis*) or 37°C (*E. coli*). Appropriate antibiotics were added to culture media when needed with ampicillin (50 μg/ml), kanamycin (50 μg/ml), chloramphenicol (20 μg/ml), and streptomycin (50 μg/ml). Non-polar deletion of *Y. pestis* KIM5 chromosomal DNA sequences *ylrC* (y3397; codons 29-515)*, ylrB* (y3399; codons 29-261), and *ylrA* (y3400; codons 23-529) was accomplished using lambda Red recombination as described by Datsenko and Wanner (2000). PCR products used to construct the gene replacement were amplified using the template plasmid pKD4 (Km^r^). The resulting PCR products were gel purified, ethanol precipitated, and resuspended in 10 μl of distilled water. *Y. pestis* KIM5 strain carrying plasmid pKD46, which encodes the Red recombinase, was induced with 0.2% L-arabinose for 2 h prior to harvest. Electrocompetent cells were electroporated with the purified PCR products. The transformations were plated onto TBA plates containing kanamycin (50 μg/ml). Plasmid pCP20, which encodes the FLP recombinase, was electroporated into the Km^r^ resistant strains to facilitate the removal of the FLP recognition target-flanked *kan* cassette and plasmid pKD46 simultaneously. Plasmid pCP20 was cured from the *Y. pestis* deletion strains by overnight growth at 39°C. Additionally, a deletion strain of the three gene sequences *ylrC-*codon 29 *to ylrA-*codon 529 was constructed using lambda Red-recombination as described above.

### Transposon Site Hybridization (TraSH)-Based Screening

A TraSH-based approach was used to map and quantify the relative abundance of the different transposon mutants in order to identify transposon mutant insertion sites that are underrepresented in the population of mutants exposed to RAW264.7 murine macrophage-like cells. The details of the mutagenesis, infection, and TraSH-screen and data analysis are described in Bartra et al. (2012).

### Infection Assays

Cell infection assays were performed essentially as described by Rosenzweig et al. (2005). RAW 264.7 murine macrophage-like cells and HeLa cells were cultured in Dubelcco's modified Eagle's medium (DMEM, Gibco) containing 10% heat-inactivated fetal bovine serum (Cellgro) at 37°C in the presence of 5% CO_2_. Cells were seeded in 24-well plates at densities of 2.5–4.0 × 10^5^ per well. Bacterial cell cultures were grown in tissue culture (TC) medium overnight at 27°C, diluted into fresh TC media and incubated with shaking at 27°C for 2 h and then shifted to 37°C for 1 h prior to being added to the cultured macrophages at a multiplicity of infection (MOI) of 30. After a 30 min attachment period, fresh DMEM medium was added to each well. At the 0 h and 8 h time points, the infected cells were lysed with 500 μl of distilled H_2_O and a portion of which was plated on TBA plates. Colony forming units (CFU) were counted after 2–3 days. For mouse infections, all procedures were in strict accordance with federal and state government guidelines for the Care and Use of Laboratory Animals of the National Institutes of Health and their use was approved for this entire study by the University of Miami institutional animal care and use committee (protocol number 15-081). Mice were infected intravenously with a total of 2000 CFU (1000 CFU each of the parental *Y. pestis* KIM5 and the isogenic Δ*yopB* or Δ*ylrABC* strains). Mice were humanely sacrificed at 48 h post infection, spleens were removed and homogenized in sterile water containing 0.05% triton X-100 by grinding through a fine wire mesh. The resulting homogenates were diluted and plated on media containing either chloramphenicol (CM) to select for the CM-resistant parental strain, as well as antibiotic-free media that allowed growth of both the parental strain and the CM-sensitive mutant strains. Two to three days later colonies were enumerated and the competition index (CI) for the parental/ Δ*yopB* and parental/Δ*ylrABC* co-infected animals was computed by dividing the CFU of the mutant by the CFU of the parental strain.

### Construction of YlrA, YlrB, and YlrC Expression Plasmids

DNA fragments used encoding YlrA, YlrB, and YlrC were PCR amplified from chromosomal DNA of *Y. pestis* KIM5. The resultant DNA fragments were digested with HindIII and BglII and inserted into HindIII- and BglII-digested pFLAG-CTC vector (Sigma-Aldrich). These vectors express full-length C-terminal FLAG-tagged YlrA-FLAG, YlrB-FLAG, and YlrC-FLAG. In addition, DNA sequences predicted to encode the YlrA, YlrB, and YlrC N-terminal T3S signal (SS) (amino acid residues 2 to 10) were deleted from each expression vector using whole plasmid PCR (Imai et al., 1991), generating plasmids pYlrA-FLAG-ΔSS, pYlrB-FLAG-ΔSS, and pYlrC-FLAG-ΔSS. Oligonucleotide pairs used were YlrASS-F and YlrASS-R, YlrBSS-F and YlrBSS-R, and YlrCSS-F and YlrCSS-R The resultant Ylr expression plasmids were transformed into *Y. pestis* KIM8Δ4 (Bartra et al., 2006).

### Construction of Vectors for β-Lactamase Translocation Studies

Expression plasmids encoding full length YlrA, YlrB, and YlrC carrying a C-terminal β*-*lactamase gene were constructed by the PCR-ligation-PCR technique (Ali and Steinkasserer, 1995). Individual *Y*. *pestis* KIM genes and upstream sequences that include each gene's ribosomal binding site were amplified by PCR from vectors encoding full-length FLAG-YlrA, FLAG-YlrB, and FLAG-YlrC, respectively, using oligonucleotides primer pairs YlrA-KpnI-F and YlrA-FL-R, YlrB-KpnI-F and YlrB-FL-R, and YlrC-KpnI-F and YlrC-FL-R. The DNA fragments encoding the Bla gene were amplified from plasmid pBSKII- using primers Bla-25-F and Bla-STOP-HindIII-R. Obtained DNA fragments were gel purified, kinased and ligated. The reaction was used for a second PCR using primers YlrA-KpnI-F and Bla-STOP-HindIII-R, YlrB-KpnI-F and Bla-STOP-HindIII-R, and YlrC-KpnI-F and Bla-STOP-HindIII-R. The resulting DNA fragments were ethanol precipitated, digested with KpnI and HindIII, and inserted into KpnI and HindIII-digested pBad_18_-Cm^r^. The resultant plasmids were transformed into a *Y. pestis* strain lacking YopE, YopJ, YopM, YopT, YopH, and YpkA.

### Yeast Studies

DNA fragments encoding YlrA, YlrB and YlrC were amplified by PCR from pFLAG-YlrA, pFLAG-YlrB, and pFLAG-YlrC, respectively, using oligonucleotides primer pairs YlrA-XhoI and YlrA-BamHI, YlrB-XhoI and YlrB-BamHI, and YlrC-XhoI and YlrC-BamHI listed in Table 2. The resultant DNA fragments were digested with BamHI and XhoI and inserted into BamHI- and XhoI-digested pREP3X. The pREP-3X vector contains the inducible *nmt1* promoter (Maundrell, 1993) and the presence of thiamine (15 μM) in the medium represses the promoter. If thiamine is not present in the media, the promoter becomes activated resulting in the transcription of the *ylr* gene cloned downstream. Plasmids generated were pREP-3X-YlrA, pREP-3X-YlrB, and pREP-3X-YlrC. In addition, site-specific mutation of three catalytic residues of the putative NEL domain of YlrA was done using the whole plasmid PCR method (Imai et al., 1991) generating plasmids pREP-3X-YlrA-C386S, pREP-3X-YlrA-R389A, and pREP-3X-YlrA-S449F. *Schizosaccharomyces pombe* h^−^ ade6-704 leu1-32 ura4-D18 was grown in Pombe Glutamate medium (PMG) at 32°C. The plasmid vector pREP3x (nmt13x Thiamine repressible promoter) was used to express the Ylr genes in fission yeast. The plasmid vectors were transformed using the lithium acetate protocol (Morita and Takegawa, 2004). The yeast strains were grown in PMG supplemented with the appropriate amino acids as well as thiamine to maintain selection for 2 days in logarithmic growth to an optical density (OD) of 0.1–0.4 at 595 nm. After growth, the cells were centrifuged at 4,000 × g, washed 3 times with distilled sterile water, and diluted for an overnight growth in media lacking thiamine. The following day, the OD of the cultures at 595 nm was measured and the cultures were diluted to an OD of 0.1. Three 10-fold serial dilutions of the cultures were made, and 5 μL of each dilution was spotted onto an agar plate with and without thiamine and grown at 32°C for 3 days.

### Secretion Assay

For standard secretion assays, *Y. pestis* strains were grown in thoroughly modified Higuchi's (TMH) medium in the presence or absence of 2.5 mM CaCl_2_ for 1 h at 27°C and then shifted to 37°C for the next 5 h as previously described (Jackson et al., 1998). FLAG-tagged Ylr proteins (pFLAG-CTC vector) were induced with 0.05 mM IPTG (isopropyl-β-D-thiogalactopyranoside) at the temperature shift.

### SDS-PAGE and Immunoblotting

Cultures of bacteria were harvested by centrifugation at 14,000 × *g* for 10 min at room temperature. Pellets of whole-cell bacteria and trichloroacetic acid (TCA)-precipitated supernatant proteins were resuspended according to the harvest OD_620_ and subjected to SDS-PAGE and immunoblotting as previously described (Jackson et al., 1998). LcrV was detected with rabbit polyclonal antisera (1:20,000) raised against the full-length LcrV proteins. FLAG-tagged proteins were detected with anti-FLAG M2 monoclonal antibody (Sigma-Aldrich) (1:1,000).

### β-Lactamase Translocation Assay

HeLa cells were seeded to a six-well plate to achieve a 40–50% confluence 1 day prior to infection. *Y. pestis* strains were grown at 27°C for 2 h in HIB with antibiotics as appropriate and then shifted to 37°C for 1 h to induce expression of T3SS proteins. At temperature shift, 0.2% L-arabinose was added to the cultures. Bacteria were added at MOI = 50 to HeLa cells. The infected cells were incubated at 37°C with 5% CO_2_. Two negative controls (without *Y. pestis* cells and *Y. pestis* with pBAD18-pYscF_1_-Bla) and a positive control were used to determine the background blue and green fluorescence. After infection for 3 h, the growth medium from the cells was removed and the cells were washed with 500 μL of 1x of GIBCO Hank's Balanced Salt Solution (HBSS). 6X CCF-AM (Invitrogen) was added to each sample to obtain a final concentration of 1X per manufacturer's instructions. The plate was covered and incubated in room temperature for 1 h. Cells were then visualized by confocal-fluorescent microscopy (Leica TCS SP5 Inverted Confocal Microscope).

## Results

### Pro-survival Activity of *Y. pestis* Leucine-Rich Proteins During Cellular Infection

**Figure 1:**
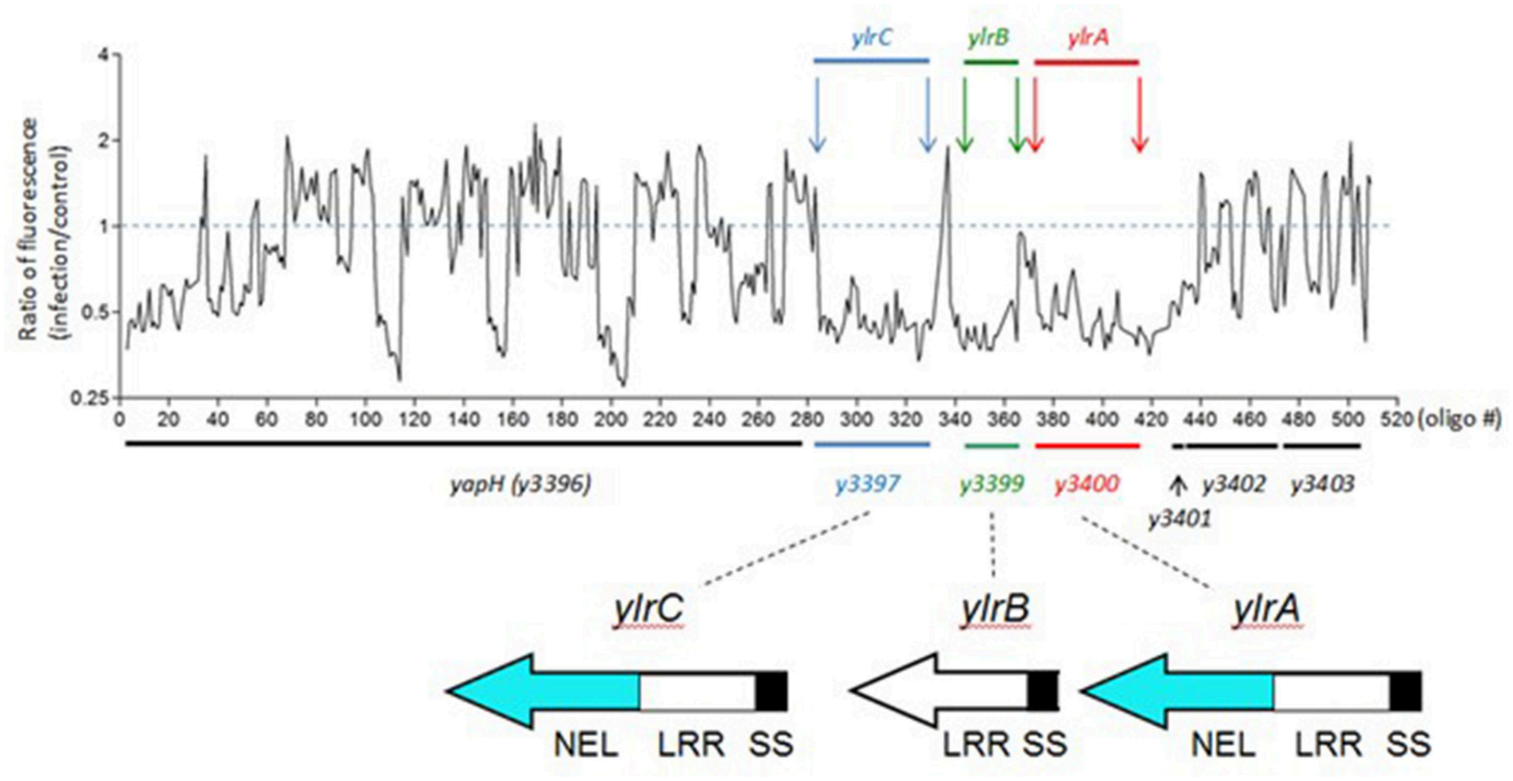
A TraSH screen indicates that the *ylrABC* locus promotes the survival of *Y. pestis* during infection. A pool of 90,000 unique *Y. pestis* transposon variants were used to infect cultured RAW 264.7 macrophages. Following infection bacterial DNA was isolated and the relative abundance of specific regions of the *Y. pestis* genome was determined. Shown in the abundance at the *ylr* locus indicating that *Y. pestis* variants possessing transposon insertion at this locus were under-represented at the termination of the infection period. Protein domains are labeled as Novel E3 Ubiquitin Ligase (NEL), Leucine-Rich Repeat (LRR), and T3SS Secretion and Translocation Signal (SS) domains.

Previously we described a transposon site hybridization (TraSH)-based screen to identify *Y. pestis* loci that are required during infection of cultured mouse macrophage-like RAW 264.7 cells (Bartra et al., 2012). From a pool of approximately 90,000 unique variants, 44 *Y. pestis* ORFs were identified that appeared to be required for pathogen survival during its interaction with the macrophage. A number of these ORFs (17) are encoded on the extrachromosomal plasmids pCD1 and pPCP1 and express components of the T3SS and Pla protease that both play critical roles in *Y. pestis* virulence (Viboud and Bliska, 2005). One such pCD1-encoded locus among this set of 17 identified ORFs, YopM, possesses Leucine-Rich Repeats (LRRs), and is associated with a variety of infection phenotypes (see Introduction). Among the 27 chromosomally-encoded pro-survival ORFs, there were three, *y3397, y3399*, and *y3400*, that were previously predicted to possess LRRs (Hu et al., 2016) and here will be designated as *Yersinia* leucine-rich repeat A (*ylrA*), B (*ylrB*), and C (*ylrC*), respectively (Figure 1). In addition to LRR-encoding sequences, these three ORFs also possess predicted secretion signals (SS) for the T3SS at their N-termini and YlrA and YlrC are additionally predicted to encode novel ubiquitin E3 ligase (NEL) domains (Wang et al., 2013; Marchler-Bauer et al., 2017).

### Defective Infection Phenotypes of Engineered Δ*ylr* Strains

**Figure 2:**
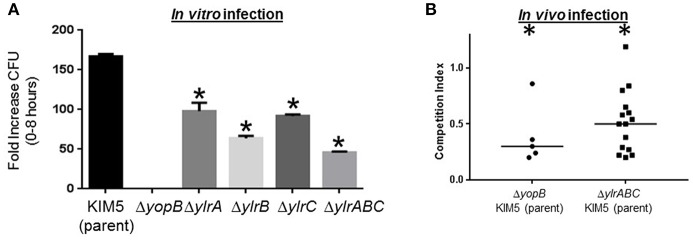
Infection defects of engineered *ylr* mutants. **(A)** Cultured RAW264.7 macrophages were infected with *Y. pestis* KIM5, Δ*yopB*, Δ*ylrA*, Δ*ylrB*, Δ*ylrC*, or Δ*ylrABC* strains at an MOI of 1. After a 30 min attachment period, unattached bacteria were removed and cell-associated bacteria (cfu) were determined by plating at the 0 and 8 h time points. Three independent wells per strain were analyzed and the average fold increase in cfu recovered for each strain over the 8 h infection period is shown (^*^P < 0.05 by Student *t*-test of a single representative experiment performed multiple times). **(B)** Mice were infected intravenously with an equal mixture of wild-type *Y. pestis* KIM5 and either the Δ*yopB* or Δ*ylrABC* strains and 2 days later mice were humanly euthanized and the relative abundance of each strain in the spleen was determined. Each data point represents an individual mouse and the medium competition index is indicated. Statistically significant reductions in splenic colonization by the mutant strain is labeled by an asterisk (*P* < 0.05 by Student *t*-test of multiple cohorts of mice in separate infections).

The aforementioned TraSH screen involves infecting macrophages with a mixture of *Y. pestis* variants followed by a quantification of under-represented genetic loci. To test whether the Ylr-encoding loci play a pro-survival function for *Y. pestis* during a single-strain infection, individual Δ*ylrA*, Δ*ylrB*, and Δ*ylrC* mutant strains were constructed and tested in a colony-forming unit (CFU)-based infection assay. Similar to what has been shown previously, the fold-increases of the parental *Y. pestis* KIM5 strain and the T3SS mutant stain (Δ*yopB*) during a 8 h infection, were 170 and 3, respectively (Figure 2A; Rosenzweig et al., 2005). The infectivity of the single gene deletion Δ*ylrA*, Δ*ylrB*, and Δ*ylrC* mutant strains, as well as the triple gene deletion Δ*ylrABC* mutant strain were intermediate between the parental and T3SS mutant strains (Figure 2A). This intermediate *in vitro* infection phenotype is similar to that observed in *Yersinia* mutants individually deleted for the YopE and YopH T3SS effectors (Bartra et al., 2001). These data validated the findings of the TraSH screen indicating that the Ylr-encoding genes promote the survival of *Y. pestis* during their interaction with macrophages.

The *Y. pestis* Δ*ylrABC* strain was tested in a mouse-based infection model to determine whether the Ylr-encoding loci promote the pathogen survival *in vivo*. A competition infection assay was used to compare the proliferation of the parental KIM5 strain to that of the mutant strain within individual mice. Accordingly, in the control experiment mice were infected with an equal mixture of the parental KIM5 and the isogenic T3SS mutant Δ*yopB* strains and after 2 days the mice were humanely sacrificed and the presence of each strain in the spleen was determined by CFU assay. In contrast to the equal ratio of the parental and Δ*yopB* stains in the “input” (i.e., the dosed inoculum), the parental strain was greatly over-represented in the “output” (i.e., the splenic homogenates derived from mice infected for 2 days) (Figure 2B). Mice similarly infected with an equal mixture of the parental KIM5 and the isogenic Δ*ylrABC* strains also yielded a statistically significant over-representation of the parental strain following 2 days of infection (Figure 2B). There was no detectable differences in growth rates between the parental KIM5 and the isogenic Δ*ylrABC* strains (Supplementary Figure 1). Collectively these data indicate that the *ylr* locus promotes *Y. pestis* infection both in cells and in mice.

### YlrA, Ylrb, and Ylrc Are Secreted by the *Y. pestis* Ysc Type III Secretion System

**Figure 3:**
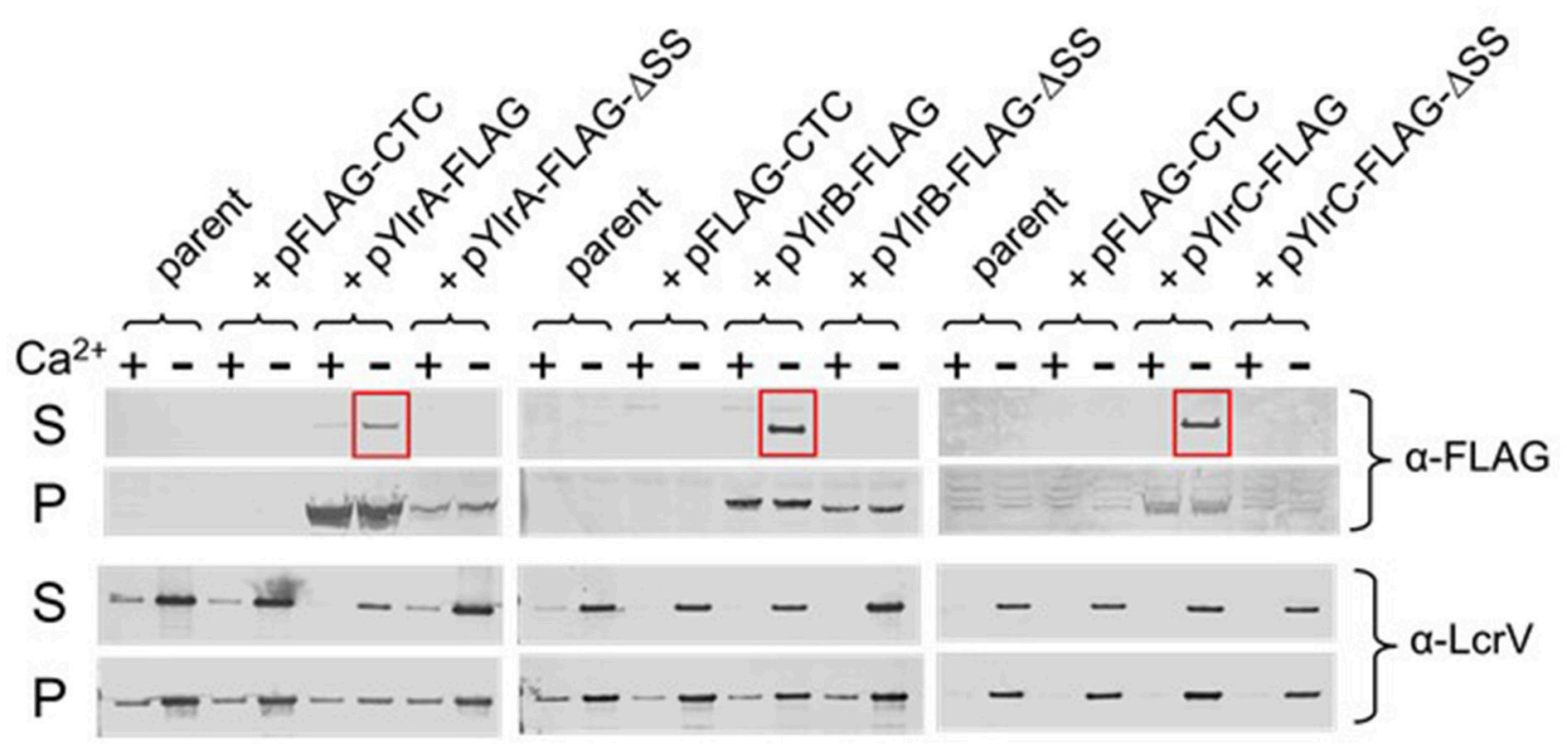
YlrA, YlrB, and YlrC are secreted by the *Y. pestis* Ysc type III secretion system. *Y. pestis* KIM8Δ4 (parent) carrying either plasmid pYlrA-FLAG, pYlrB-FLAG, or pYlrC-FLAG as well as plasmids deleted for sequences encoding the predicted Ylr secretion signals (ΔSS) were grown for 5 h in the presence (+) and absence (–) of 2.5 mM Ca^2+.^ Cell pellet (P) and culture supernatant (S) fractions were evaluated by SDS-PAGE and immunoblot analysis with anti-FLAG and anti-LcrV (control) antibodies.

Sequence analysis of the *ylrA, ylrB*, and *ylrC* genes indicated that they each potentially encode at their amino-terminus a recognition sequence for the type III secretion system (T3SS) (Hu et al., 2016). To determine if the YlrA, YlrB, and YlrC proteins are secreted by the T3SS, expression vectors containing full-length C-terminal FLAG-tagged proteins were constructed. In addition, DNA sequences predicted to encode YlrA, YlrB, and YlrC N-terminal T3SS signal residues 2 to 10 were deleted from each of the expression vectors. Generated plasmids were placed in *Y. pestis* KIM8Δ4, a strain containing a modified pCD1 virulence plasmid that contains all of the genes necessary to assemble a functional T3SS but lacks all other effector proteins (Bartra et al., 2006). The strains were tested for expression and secretion in the presence and absence of 2.5 mM of calcium and bacterial pellet and supernatant were analyzed. FLAG-tagged YlrA, YlrB, and YlrC were expressed in the presence and absence of calcium, but were secreted only in the absence of calcium (Figure 3; *red boxes*; and Data Sheet 1). There was no secretion of the YlrA, YlrB, and YlrC proteins when the putative T3SS signal of each protein was deleted. Finally, there was no secretion of YlrA-FLAG, YlrB-FLAG, or YlrC-FLAG detected by *Y. pestis* strain KIM8 (pCD1-) or *Y. pestis* Δ*yscF*, both of which are unable to assemble a functional Ysc injectisome (Supplementary Figures 2–4). Together, these results demonstrate that YlrA, YlrB and YlrC are T3S substrates and that they are recognized and secreted by the *Y. pestis* T3SS.

### YlrB Is Translocated Into Mammalian Cells

**Figure 4:**
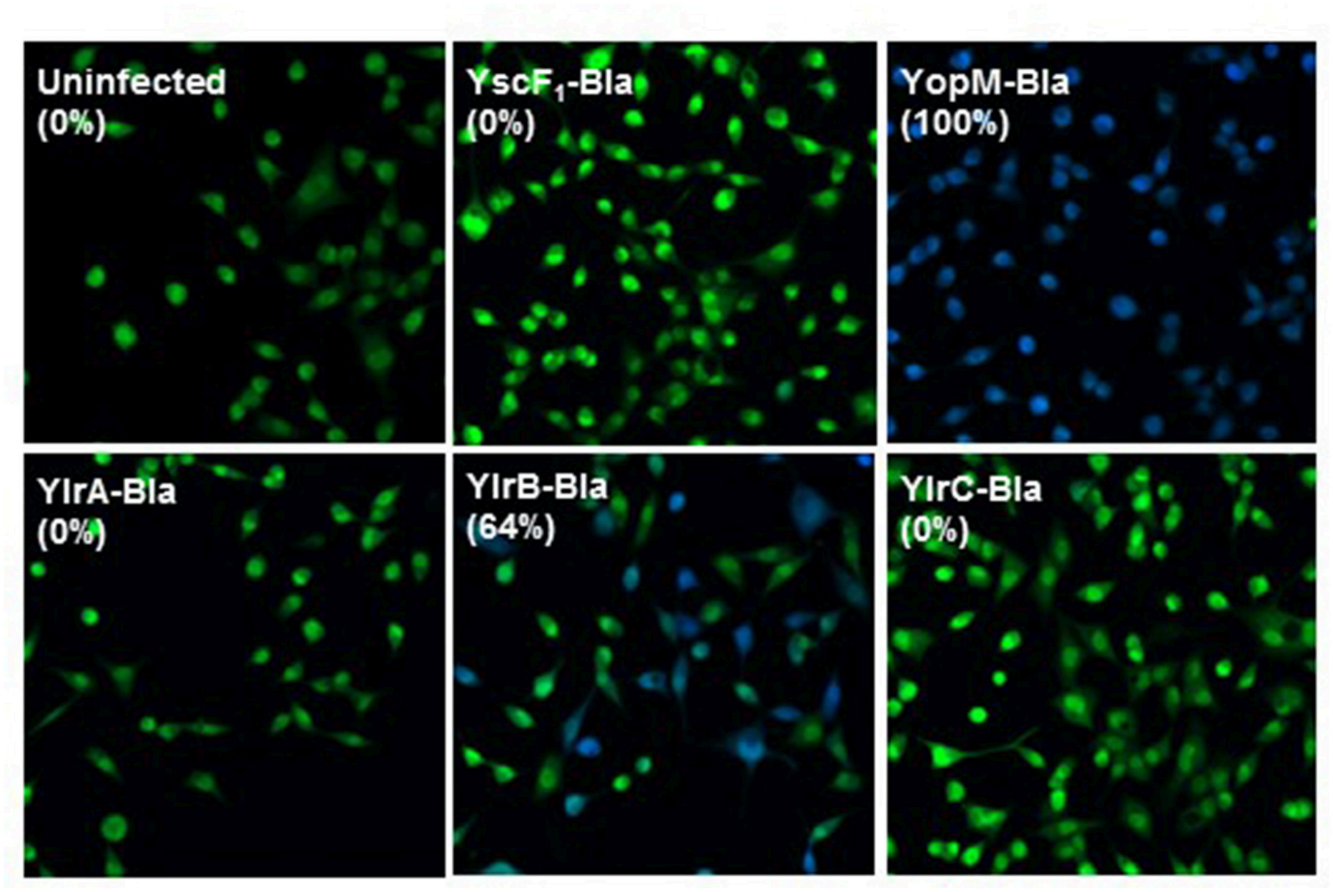
YlrB is translocated into eukaryotic cells. HeLa cells were infected with Yp589 (*Y. pestis* strain with deletion of YopE, YopJ, YopM, SycT, and YpKA) carrying YscF_1_-Bla (possessing the *yscF* 5′ UTR and the initiation codon), pMM85 YopM-Bla, YlrA-Bla, YlrB-Bla, or YlrC-Bla and were then incubated at 37°C for 3 h. CCF2-AM was added to HeLa cells and visualized by fluorescence microscopy. HeLa cells infected with the strain carrying the YlrB-Bla were positive for cytoplasmic β-lactamase activity (blue fluorescence), indicating that these cells had been injected with YlrB-Bla. Shown is a single representative experiment performed multiple times.

A β-lactamase reporter system was used to determine whether Ylr proteins are translocated into cultured cells during infection (Marketon et al., 2005). Upon diffusion into cultured cells, CCF2-AM is cleaved into the membrane-impermeable CCF2 by endogenous cytoplasmic esterases, emitting a green fluorescence. When β-lactamase fusion proteins are injected into the cells, CCF2 can be cleaved further, changing the fluorescent emission to blue (Charpentier and Oswald, 2004). Cells that were either uninfected or infected with a *Y. pestis* strain expressing YscF_1_-Bla, which is expressed but not secreted by the T3SS, retained their initial green fluorescence (Figure 4). In striking contrast, essentially all CCF2-labeled cells infected with a *Y. pestis* strain expressing YopM-Bla, which, as discussed in the Introduction, is a plasmid-encoded virulence factor, fluoresce blue indicating that YopM-Bla is readily translocated into the infected cell cytosol. Cells infected with *Y. pestis* strains expressing either YlrA-Bla or YlrC-Bla retained their green fluorescence indicating that these proteins were not detectably translocated as assayed by this method despite being readily expressed as assayed by western blotting (Figure 4; Supplementary Figure 5). In contrast, blue fluorescing cells were readily detected following infection with a *Y. pestis* strain expressing YlrB-Bla. These show that the YlrB is translocated by *Y. pestis* during infection.

### Intracellular Activity of Ylr

**Figure 5:**
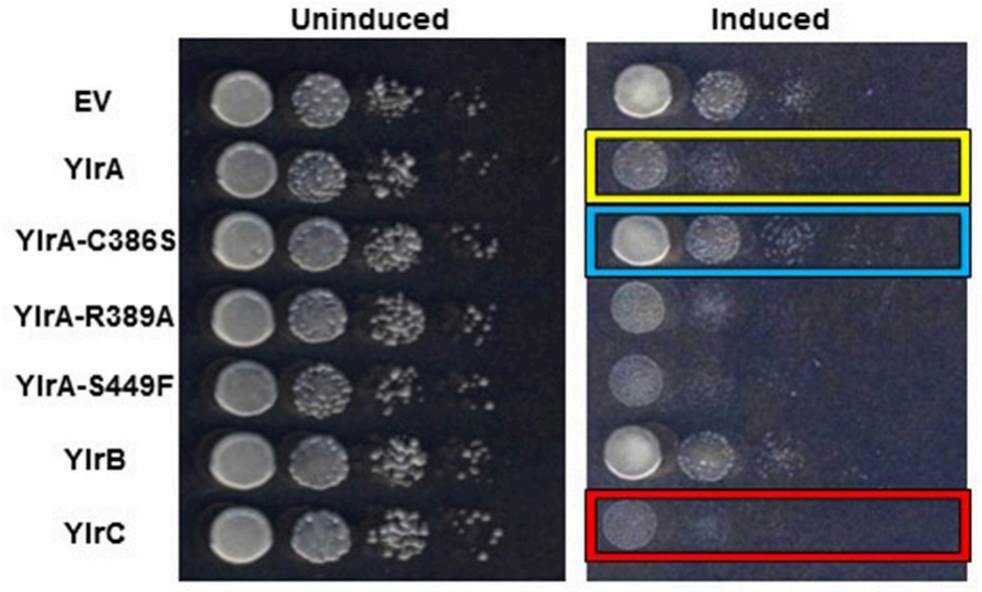
YlrA and YlrC, but not YlrB, disrupt yeast growth. Yeast transformed with either an empty vector (EV) control plasmid or the indicated Ylr proteins under the control of an inducible promoter. Comparable growth was observed when strains were plated on non-inducing media (*left panel*). However, when strains were plated on inducing media (*right panel*), the YlrA- and YlrC-expressing strains (yellow and red boxes, respectively) displayed a growth defect compared to the EV and YlrB-expressing strains. Also, mutation of the putative catalytic cysteine residue of the NEL domain of YlrA reversed the disruptive effect of YlrA on yeast growth (blue box); whereas mutation of the other residues did not have any effect. Shown is the result of a single experiment performed three times with similar outcomes.

Previous studies of the *Yersinia* Yop virulence factors have shown a robust correlation between their growth inhibition in yeast and their activity in mammalian cells (Lesser and Miller, 2001; Wiley et al., 2009). Yeast provide a relatively simple genetic model system for the study of the eukaryotic genes and is based on the premise that yeast share many molecular, genetic, and biochemical features with mammalian cells (Zhao and Lieberman, 1995). Accordingly, the fission yeast *Schizosaccharomyces pombe* was transformed with either an empty vector control plasmid or inducible expression plasmids encoding either YlrA, YlrB, or YlrC and plated either on media in which the Ylr proteins were not expressed (“uninduced”) or on media in which Ylr expression occurs (“induced”). All *S. pombe* transformant strains grew equally well on uninduced media (Figure 5). However, on induced media, there was a marked differences between the relatively rapid growth of the empty vector and YlrB transformants (*rows 1 and 6*) and that of the relatively poorer growth of the YlrA and YlrC transformants (*rows 2 and 7*). Previous work has shown that IpaH effectors and SspH2 have a conserved catalytic cysteine residue that is absolutely required for ubiquitin ligase activity (Rohde et al., 2007; Quezada et al., 2009). To determine if this residue and other conserved residues of the NEL domain of YlrA will affect its growth-inhibiting activity in yeast, these residues were substituted generating plasmids expressing YlrA-C386S, YlrA-R389A, and YlrA-S449F. There was a clear reduction in growth inhibition for the *S. pombe* strain expressing the YlrA-C386S variant compared to the strain expressing wild-type YlrA (Figure 5, *rows 2 and 3*). In contrast, there was no detectable loss of growth inhibitory activity in the *S. pombe* strains expressing either the YlrA-R389A or YlrA-S449F (*rows 4 and 5*). These data suggest that the ubiquitin ligase activity of YlrA is required for its growth inhibitory activity in eukaryotic cells.

## Discussion

In this study, we demonstrate that the *Y. pestis* chromosomally-encoded proteins YlrA, YlrB, and YlrC are secreted and recognized by the T3SS and are required for the optimal survival of this pathogen both in the presence of cells as well as in mice. Although many chromosomally-encoded *Y. pestis* virulence factors have been described, to the best of our knowledge the Ylr are the first chromosomally-encoded virulence factors secreted by the pCD1-encoded T3SS.

YlrA, YlrB, and YlrC are leucine rich repeat proteins (LRR) with (YlrA and YlrC) or without (YlrB) an E3 ligase domain (NEL). The Ylr proteins belong to a diverse group of molecules distinguished by a consensus sequence consisting predominantly of leucines, hence the term leucine-rich repeat. Similar proteins are found in other Gram-negative bacteria that possess T3SSs, including *Yersinia* (YopM) (Kobe and Deisenhofer, 1994)*, Shigella* (IpaH family proteins) (Ashida et al., 2007)*, Salmonella* (SspH1, SspH2 and SlrP) (Miao et al., 1999; Tsolis et al., 1999; Bernal-Bayard et al., 2010) and other bacterial pathogens. In addition to possessing detectable membrane-penetrating activity, LRR domains offer adaptable structural frameworks that mediate protein-protein interactions, coupling enzymatic domains to substrate-binding domains, function to determine substrate specificity, and to suppress the enzymatic activity of the NEL domain prior to substrate binding (Kobe and Kajava, 2001; Quezada et al., 2009; Rüter et al., 2010). The NEL domains function as ubiquitin E3 ligases which mimic the activities of host E3 ubiquitin ligases and ubiquitinate specific target proteins (Rohde et al., 2007; Quezada et al., 2009). YlrA and YlrC contain an N-terminal LRR domain and a predicted C-terminal novel E3 ligase domain (NEL) (Hu et al., 2016). E3 ligase activity has been demonstrated for the *Shigella* effectors IpaH4.5 and IpaH9.8 and *Salmonella* effectors SspH1, SspH2, and SlrP (Rohde et al., 2007; Singer et al., 2008; Zhu et al., 2008; Bernal-Bayard and Ramos-Morales, 2009; Quezada et al., 2009). Bacterial E3 ubiquitin ligases transported into host cells mimic the activities of host E3 ubiquitin ligases and ubiquitinate specific target proteins. For example, IpaH 4.5 appears to dampen the innate immune system of human cells by inhibiting nuclear factor KB (NF-KB) signaling in response to an intracellular infection with *Shigella* (Ashida et al., 2010). The *Salmonella* virulence effector SlrP targets the mammalian thioredoxin-1 (Trx) leading to ubiquitination of Trx and causes a decrease in its redox activity (Bernal-Bayard and Ramos-Morales, 2009).

We have demonstrated that YlrB is translocated into the eukaryotic cell, whereas there was no evidence of translocation of the YlrA and YlrC using a β-lactamase reporter system. These results are difficult to interpret. Both full-length YlrA and YlrC are expressed and secreted, albeit at lower levels than YlrB (see Figure 3 and Supplementary Figure 5); therefore we expect them to be translocated as well. In addition, the fact that they contain an E3 ligase domain suggests that these proteins work inside the eukaryotic cell. Lack of detectable translocation in our experiments could be due to the large size of the proteins (~600 amino acids). Further experiments are needed using smaller-sized constructs expressing the translocation domains of both proteins. The most compelling data we present that YlrA and YlrC actually function within eukaryotic cells is the fact that they both disrupt the growth of the fission yeast *S. pombe*. Furthermore, to test if the conserved catalytic cysteine residue in YlrA is important to the activity of the protein, it was replaced with a serine residue. This resulted to the loss of the growth-inhibiting activity of YlrA. We speculate that the deleterious effect of YlrA and YlrC is due to the activity of their NEL domain. In a study using a yeast model, Rohde et al. (2007) have shown that *S. flexneri* IpaH9.8 inhibit yeast pheromone response signaling by ubiquitination and by promoting proteasome-dependent destruction of the mitogen-activated protein kinase kinase (MAPKK) Ste7. It was also demonstrated in the same study that the cysteine residue conserved in the NEL domain of all IpaH family members is required for the IpaH9.8 activities in yeast. The *Salmonella* effector SspH2 also has a conserved cysteine residue (Quezada et al., 2009). This catalytic cysteine residue and all other NEL active site residues are absolutely conserved between YlrA, YlrC and SspH2. We conclude that both YlrA and YlrC are strong candidates to join the list of bacterial virulence proteins that target the mammalian ubiquitination system.

To date, little is known about the leucine-reach repeat proteins in *Yersinia* with the exception of YopM. Miao et al. (1999) described three leucine-rich repeat open reading frames (ORFs) in *Y. pestis* that share significant homology with SspH2. These ORFs seem to be *ylrA, ylrB* and *ylrC*, since the ORFs were described to be present in an operon-like structure in the chromosome. Later, Evdokimov et al. (2001) described that these three ORFs share similar structure with *Salmonella* effectors SlrP, SspH1, SspH2; *Shigella* effector IpaH; and *Yersinia* effector YopM. Chou et al. (2012) PCR amplified *Y. pestis* y3400 (*ylrA*), expressed and purified the previously uncharacterized protein in *E. coli* and demonstrated that the purified protein possessed constitutive, not autoinhibited, ubiquitin E3 ligase activity. Finally, Soundararajan et al. (2011) were the first to identify and analyze the structure-function relation of a protein which appears to be the protein product of *y3397* (YlrC) (McPhee and Bliska, 2011). More systematic studies are needed to characterize the expression, secretion, translocation, molecular functions and host target of these novel effector proteins. Research on these *ylr* gene products will lead to a better understanding of *Y. pestis* pathogenesis and possibly to the identification of the proteins as novel vaccine candidates or potential targets for therapeutics.

In conclusion, the *ylrA, ylrB*, and *ylrC* gene products represent the first chromosome-encoded T3S effector proteins of *Y. pestis* and are required for the optimal survival of this pathogen in the presence of macrophages. The gene products share significant similarity with T3S effector proteins from other bacterial pathogens and hence they are strong candidates to join the list of bacterial virulence proteins.

## Author Contributions

SS, CL, XG, WB, ZL, GP, and KS planned and performed the majority of the experiments. HB and RH provided technical assistance. LQ analyzed the results of the TraSH screen. SS, CL, and KS wrote the manuscript.

### Conflict of Interest Statement

The authors declare that the research was conducted in the absence of any commercial or financial relationships that could be construed as a potential conflict of interest.
