# Supplementary material for: Chromosomally-Encoded Yersinia pestis Type III Secretion Effector Proteins Promote Infection in Cells and in Mice
Source: Front Cell Infect Microbiol. 2019 Feb 22;9:23. doi: 10.3389/fcimb.2019.00023 (PMC6396649; doi:10.3389/fcimb.2019.00023)
Supplement: Supplementary file 1 [file Presentation_1.PPTX]

## Slide 1
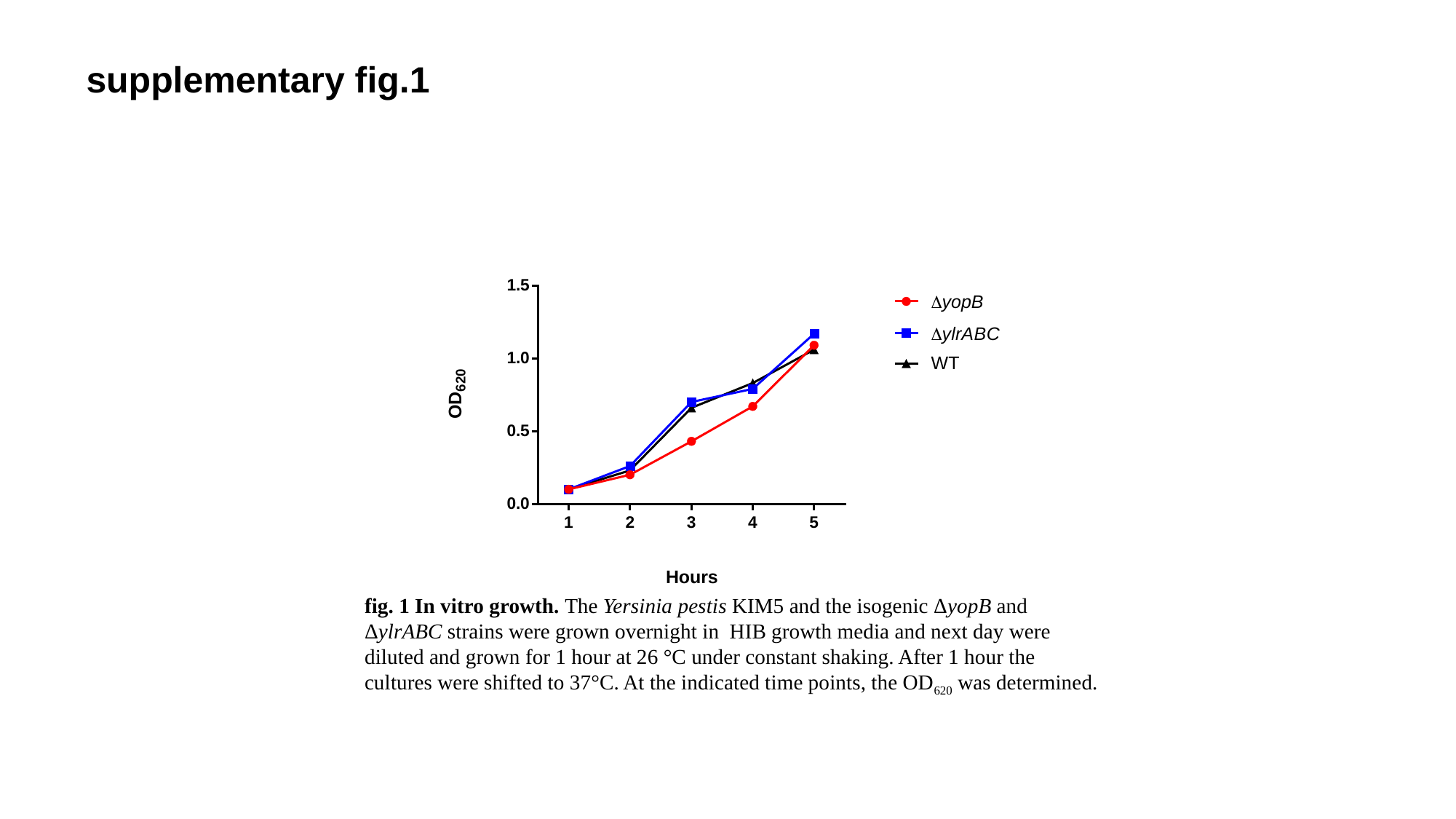

supplementary fig.1
fig. 1 In vitro growth. The Yersinia pestis KIM5 and the isogenic ΔyopB and ΔylrABC strains were grown overnight in HIB growth media and next day were diluted and grown for 1 hour at 26 °C under constant shaking. After 1 hour the cultures were shifted to 37°C. At the indicated time points, the OD620 was determined.

## Slide 2
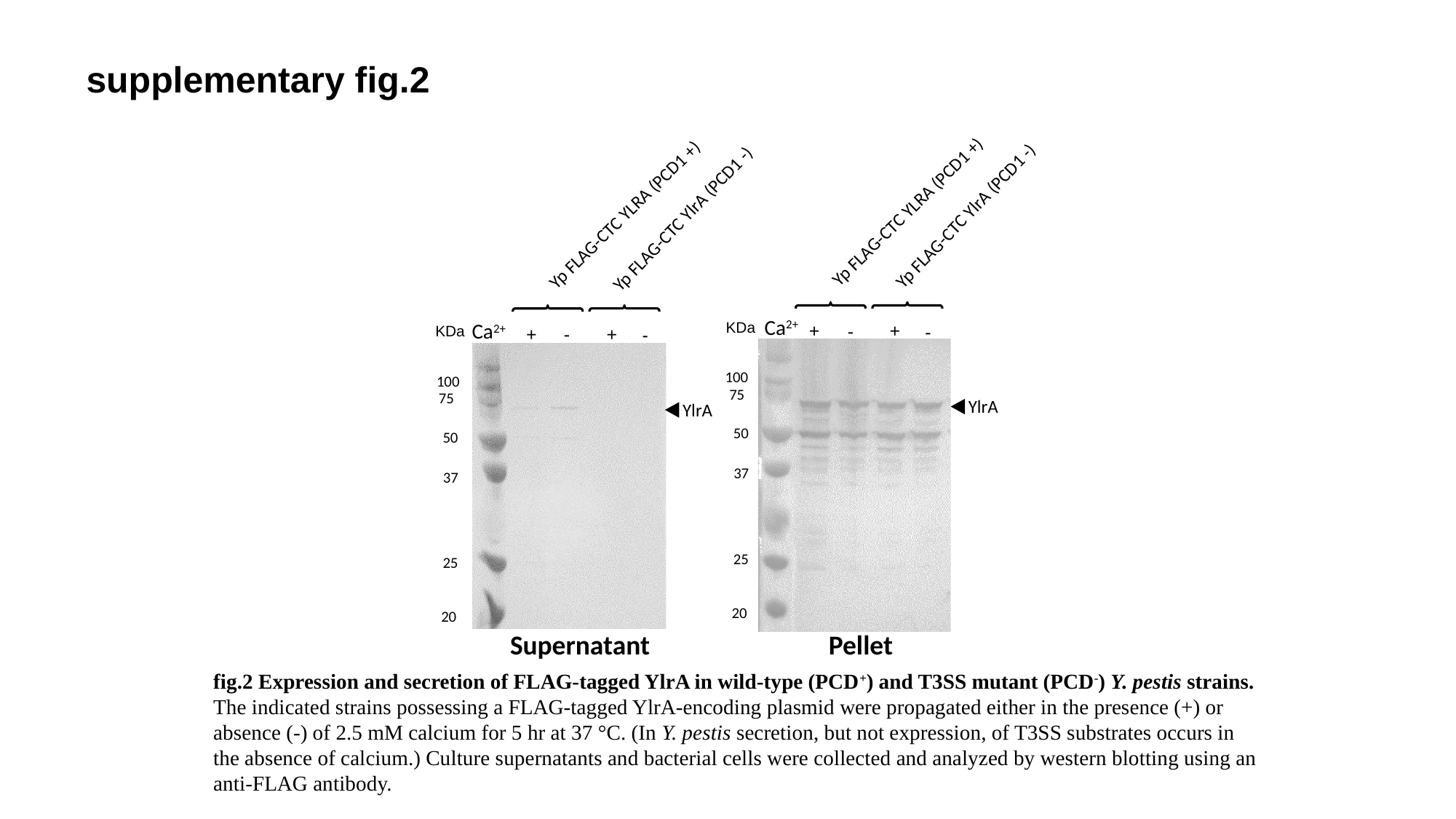

supplementary fig.2
Yp FLAG-CTC YLRA (PCD1 +)
Yp FLAG-CTC YlrA (PCD1 -)
Ca2+
KDa
+
+
-
-
 100
 75
50
37
25
20
YlrA
Yp FLAG-CTC YLRA (PCD1 +)
Yp FLAG-CTC YlrA (PCD1 -)
Ca2+
KDa
+
+
-
-
 100
 75
YlrA
50
37
25
20
Supernatant Pellet
fig.2 Expression and secretion of FLAG-tagged YlrA in wild-type (PCD+) and T3SS mutant (PCD-) Y. pestis strains. The indicated strains possessing a FLAG-tagged YlrA-encoding plasmid were propagated either in the presence (+) or absence (-) of 2.5 mM calcium for 5 hr at 37 °C. (In Y. pestis secretion, but not expression, of T3SS substrates occurs in the absence of calcium.) Culture supernatants and bacterial cells were collected and analyzed by western blotting using an anti-FLAG antibody.

## Slide 3
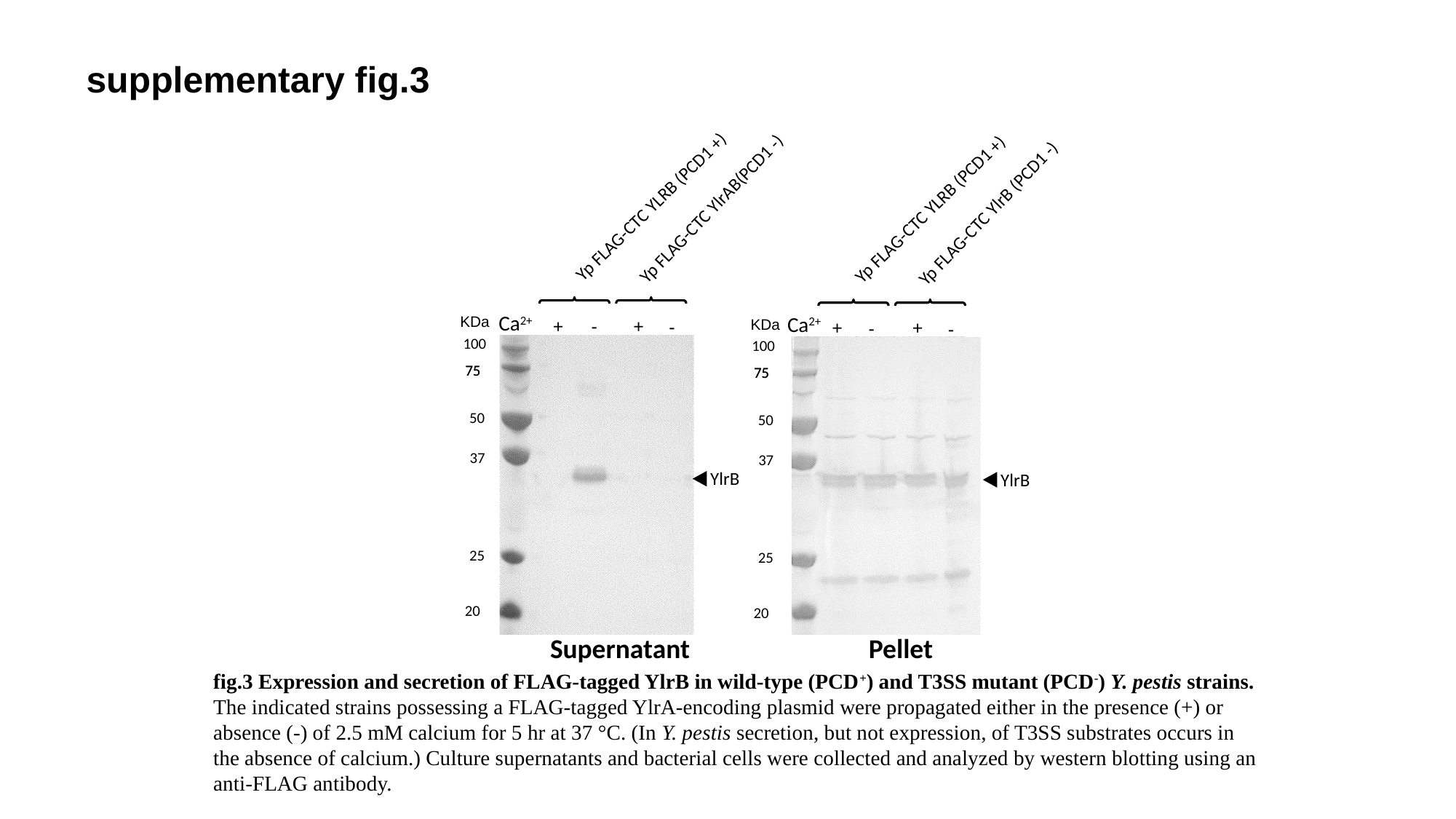

supplementary fig.3
Yp FLAG-CTC YlrAB(PCD1 -)
Yp FLAG-CTC YLRB (PCD1 +)
Ca2+
KDa
+
+
-
-
 100
 75
 75
50
37
YlrB
25
20
Yp FLAG-CTC YLRB (PCD1 +)
Yp FLAG-CTC YlrB (PCD1 -)
Ca2+
KDa
+
+
-
-
 100
 75
 75
50
37
YlrB
25
20
Supernatant Pellet
fig.3 Expression and secretion of FLAG-tagged YlrB in wild-type (PCD+) and T3SS mutant (PCD-) Y. pestis strains. The indicated strains possessing a FLAG-tagged YlrA-encoding plasmid were propagated either in the presence (+) or absence (-) of 2.5 mM calcium for 5 hr at 37 °C. (In Y. pestis secretion, but not expression, of T3SS substrates occurs in the absence of calcium.) Culture supernatants and bacterial cells were collected and analyzed by western blotting using an anti-FLAG antibody.

## Slide 4
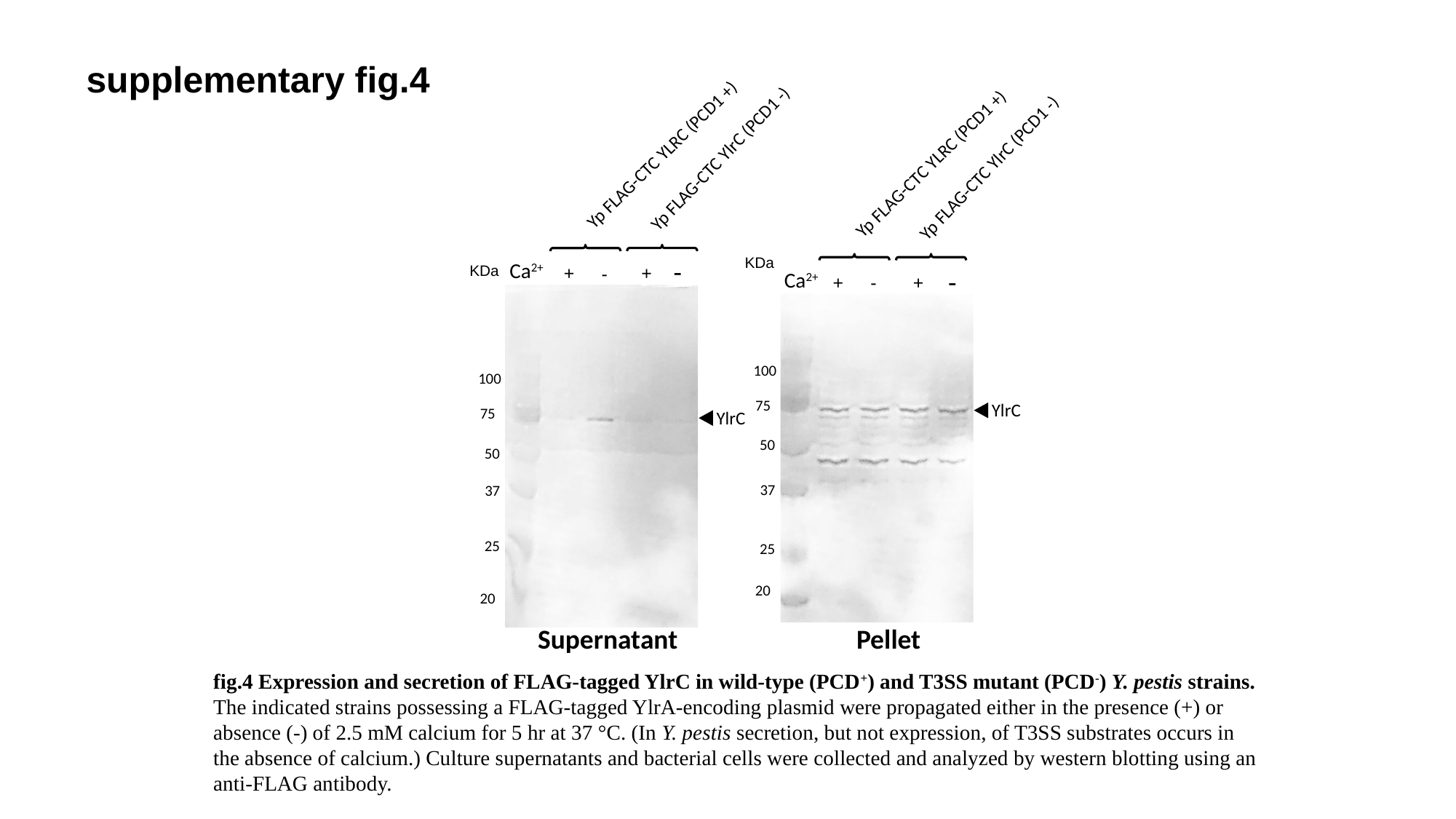

Yp FLAG-CTC YLRC (PCD1 +)
Yp FLAG-CTC YlrC (PCD1 -)
-
Ca2+
KDa
+
+
-
 100
 75
YlrC
50
37
25
20
supplementary fig.4
Yp FLAG-CTC YLRC (PCD1 +)
Yp FLAG-CTC YlrC (PCD1 -)
KDa
-
Ca2+
+
+
-
 100
 75
YlrC
50
37
25
20
Supernatant Pellet
fig.4 Expression and secretion of FLAG-tagged YlrC in wild-type (PCD+) and T3SS mutant (PCD-) Y. pestis strains. The indicated strains possessing a FLAG-tagged YlrA-encoding plasmid were propagated either in the presence (+) or absence (-) of 2.5 mM calcium for 5 hr at 37 °C. (In Y. pestis secretion, but not expression, of T3SS substrates occurs in the absence of calcium.) Culture supernatants and bacterial cells were collected and analyzed by western blotting using an anti-FLAG antibody.

## Slide 5
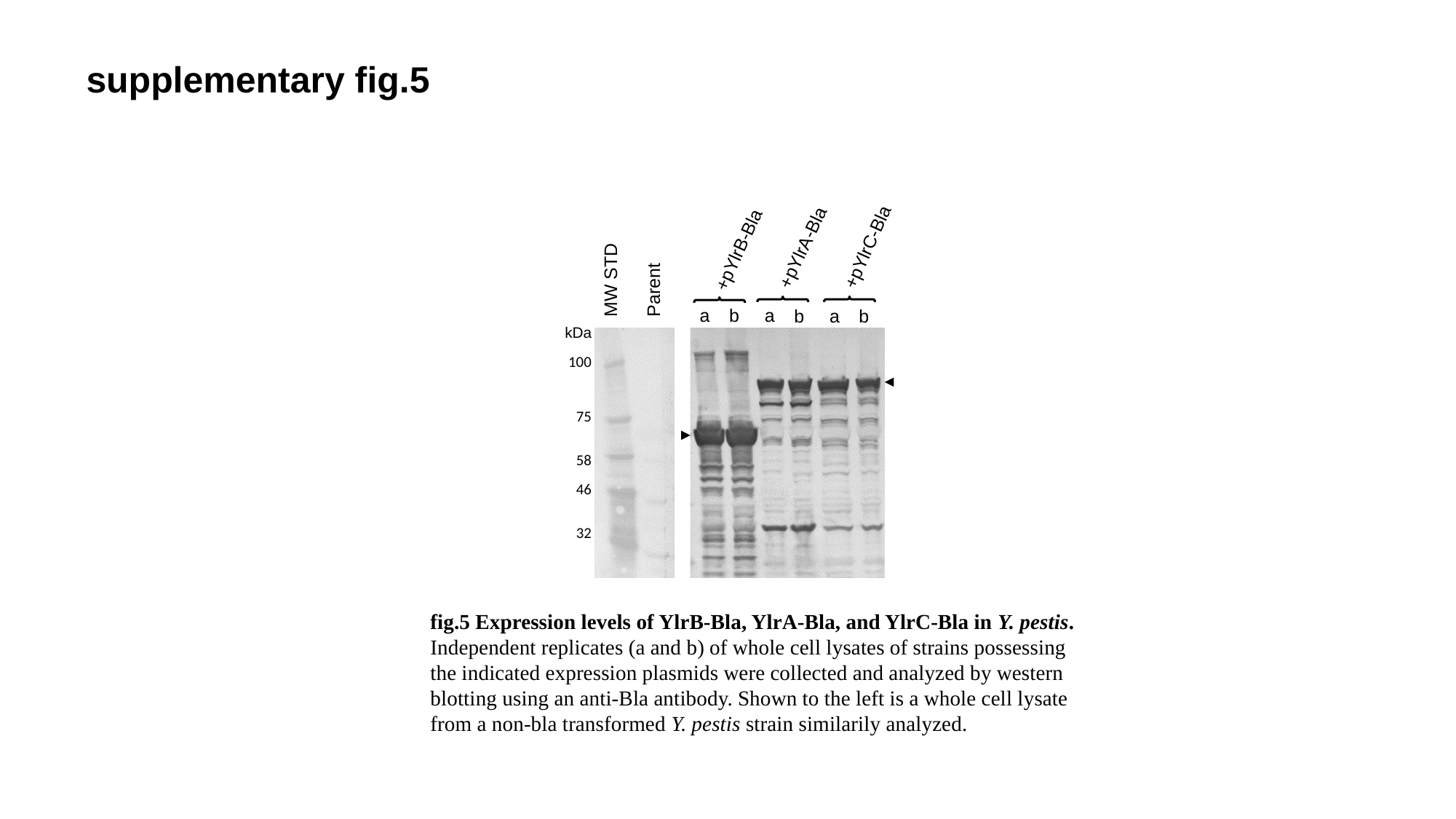

supplementary fig.5
+pYlrC-Bla
+pYlrA-Bla
+pYlrB-Bla
MW STD
Parent
a
b
a
b
a
b
kDa
100
75
58
46
32
fig.5 Expression levels of YlrB-Bla, YlrA-Bla, and YlrC-Bla in Y. pestis. Independent replicates (a and b) of whole cell lysates of strains possessing the indicated expression plasmids were collected and analyzed by western blotting using an anti-Bla antibody. Shown to the left is a whole cell lysate from a non-bla transformed Y. pestis strain similarily analyzed.
