# Supplementary material for: Chromosomally-Encoded Yersinia pestis Type III Secretion Effector Proteins Promote Infection in Cells and in Mice
Source: Front Cell Infect Microbiol. 2019 Feb 22;9:23. doi: 10.3389/fcimb.2019.00023 (PMC6396649; doi:10.3389/fcimb.2019.00023)
Supplement: Supplementary file 2 [file Data_Sheet_1.PDF]

LRR1 whole cell

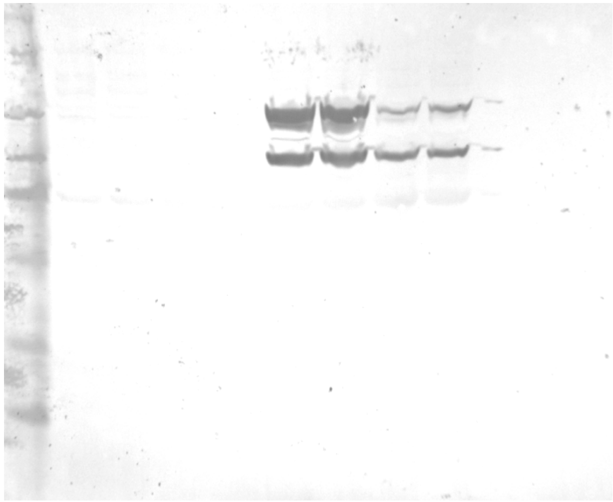

1 2 3 4 5 6 7 8

LRR1 supernatant

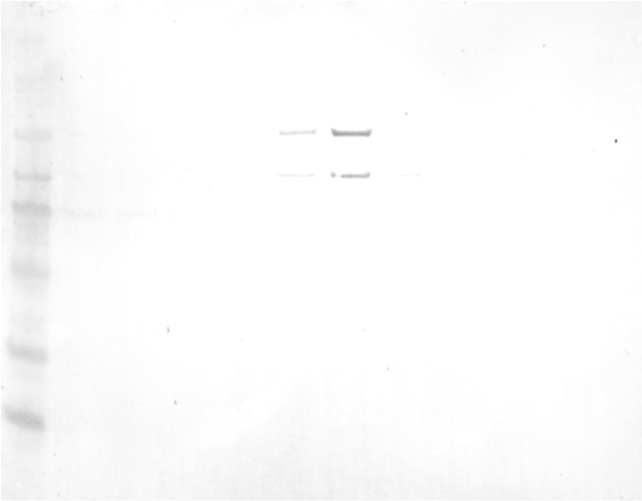

1 2 3 4 5 6 7 8

1. Yp 769 +Ca^{2+} (D28x mini pCD1)
2. Yp 769 –Ca^{2+}
3. Yp 769 + pflag-CTC +Ca^{2+}
4. Yp 769 + pflag-CTC -$Ca^{2\ +}$
5. Yp 769 + pYlrA-flag-CTC +Ca^{2+}
6. Yp 769 + pYlrA-flag-CTC -Ca^{2+}
7. Yp 769 + pYlrAΔSS-flag-CTC +Ca^{2+}
8. Yp 769 + pYlrAΔSS-flag-CTC +Ca^{2}

1 mM IPTG
α-FLAG monoclonal

LRR2 whole cell

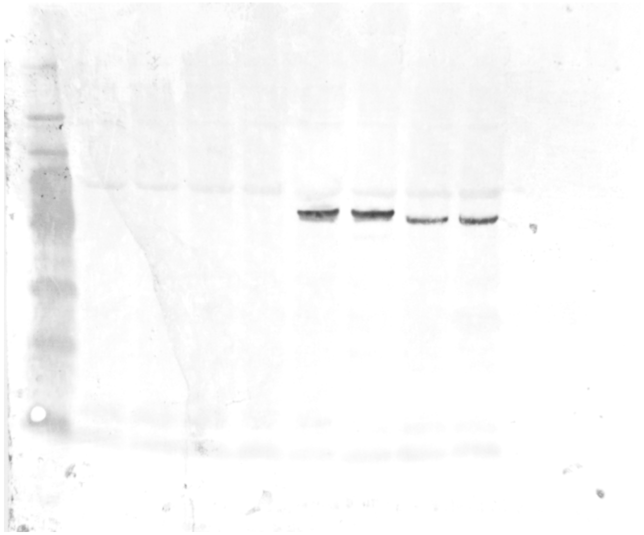

1 2 3 4 5 6 7 8

LRR2 supernatant

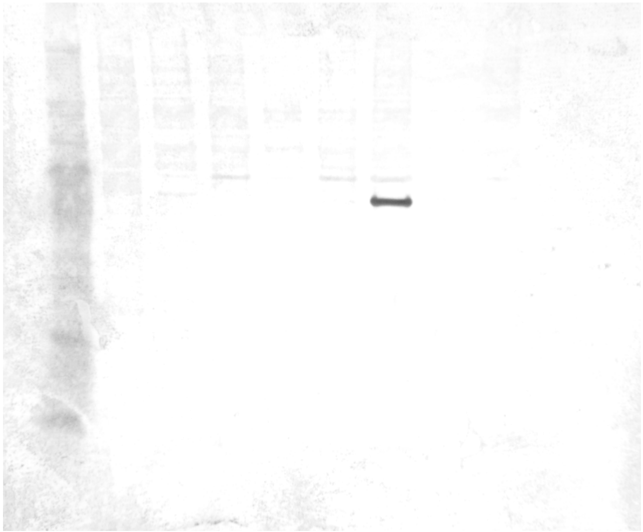

1 2 3 4 5 6 7 8

1. Yp 769 +Ca^{2+} (D28x mini pCD1)
2. Yp 769 –Ca^{2+}
3. Yp 769 + pflag-CTC +Ca^{2+}
4. Yp 769 + pflag-CTC -$Ca^{2\ +}$
5. Yp 769 + pYlrB-flag-CTC +Ca^{2+}
6. Yp 769 + pYlrB-flag-CTC -Ca^{2+}
7. Yp 769 + pYlrBΔSS-flag-CTC +Ca^{2+}
8. Yp 769 + pYlrBΔSS-flag-CTC +Ca^{2}

1 mM IPTG
α-FLAG monoclonal

LRR3 whole cell

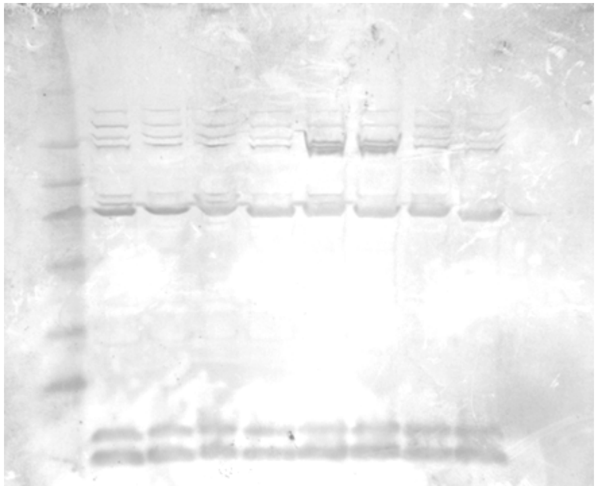

1 2 3 4 5 6 7 8

LRR3 supernatant

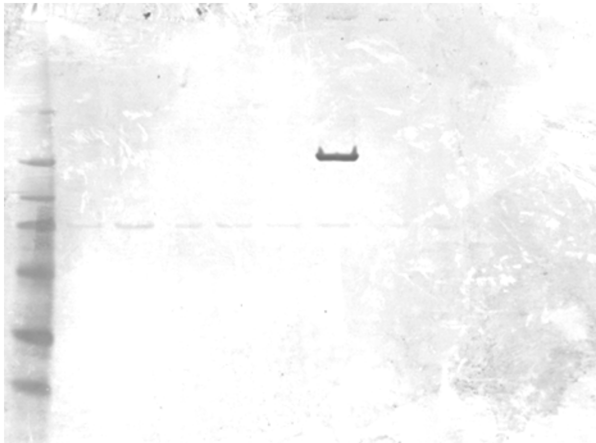

1 2 3 4 5 6 7 8

1. Yp 769 +Ca^{2+} (D28x mini pCD1)
2. Yp 769 –Ca^{2+}
3. Yp 769 + pflag-CTC +Ca^{2+}
4. Yp 769 + pflag-CTC -Ca^{2+}
5. Yp 769 + pYlrC-flag-CTC +Ca^{2+}
6. Yp 769 + pYlrC-flag-CTC -Ca^{2+}
7. Yp 769 + pYlrCΔSS-flag-CTC +Ca^{2+}
8. Yp 769 + pYlrCΔSS-flag-CTC +Ca^{2}

1 mM IPTG
α-FLAG monoclonal

LRR1 whole cell

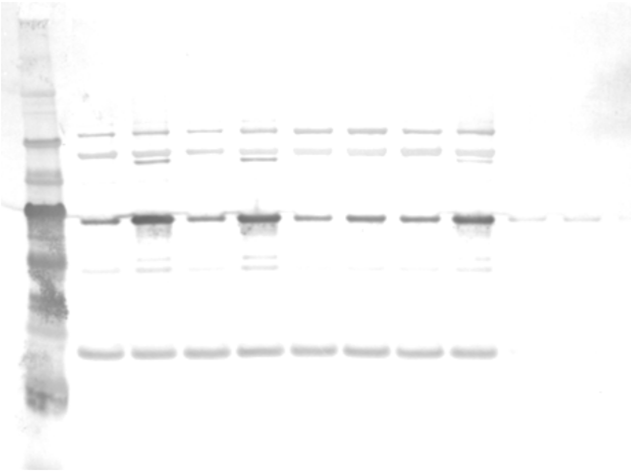

1 2 3 4 5 6 7 8

LRR1 supernatant

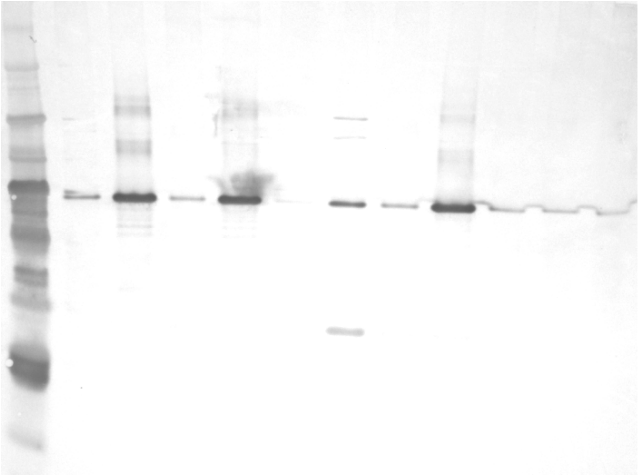

1 2 3 4 5 6 7 8

1. Yp 769 +Ca^{2+} (D28x mini pCD1)
2. Yp 769 –Ca^{2+}
3. Yp 769 + pflag-CTC +Ca^{2+}
4. Yp 769 + pflag-CTC -Ca^{2+}
5. Yp 769 + pYlrA-flag-CTC +Ca^{2+}
6. Yp 769 + pYlrA-flag-CTC -Ca^{2+}
7. Yp 769 + pYlrAΔSS-flag-CTC +Ca^{2+}
8. Yp 769 + pYlrAΔSS-flag-CTC +Ca^{2}

1 mM IPTG
α-LcrV monoclonal

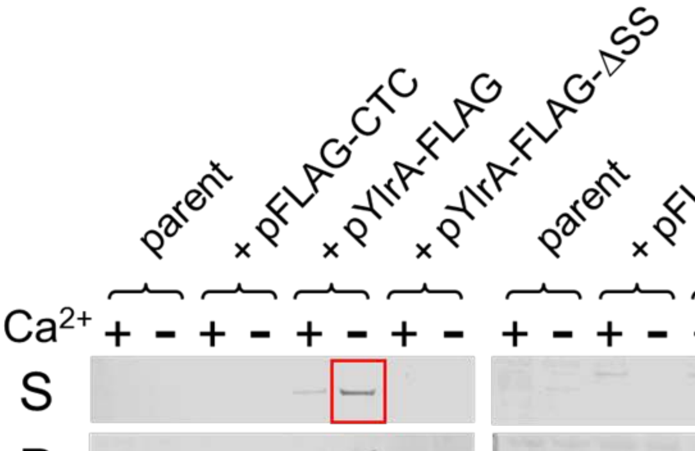

LRR2 whole cell

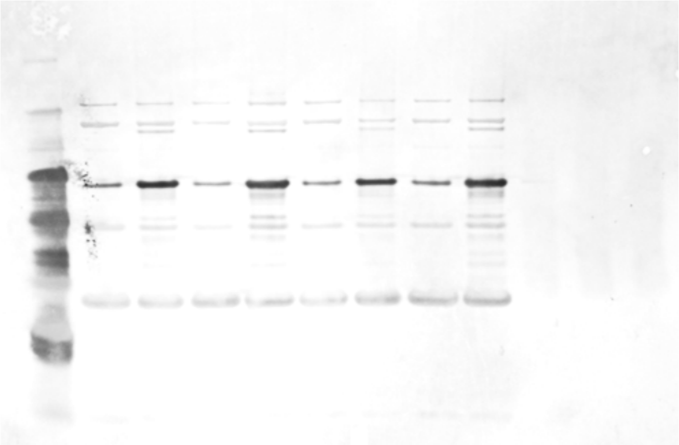

1 2 3 4 5 6 7 8

LRR2 supernatant

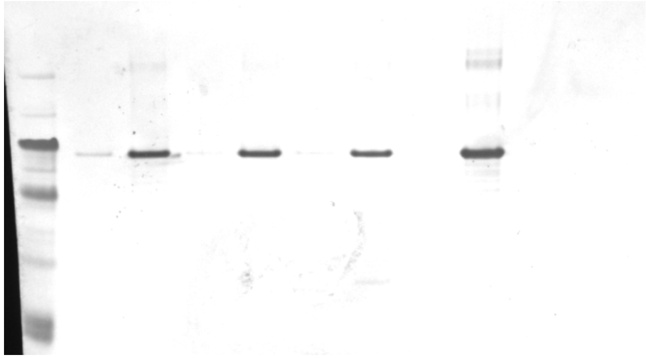

1 2 3 4 5 6 7 8

1. Yp 769 +Ca^{2+} (D28x mini pCD1)
2. Yp 769 –Ca^{2+}
3. Yp 769 + pflag-CTC +Ca^{2+}
4. Yp 769 + pflag-CTC -$Ca^{2\ +}$
5. Yp 769 + pYlrB-flag-CTC +Ca^{2+}
6. Yp 769 + pYlrB-flag-CTC -Ca^{2+}
7. Yp 769 + pYlrBΔSS-flag-CTC +Ca^{2+}
8. Yp 769 + pYlrBΔSS-flag-CTC +Ca^{2}

1 mM IPTG
α-LcrV monoclonal

LRR3 whole cell

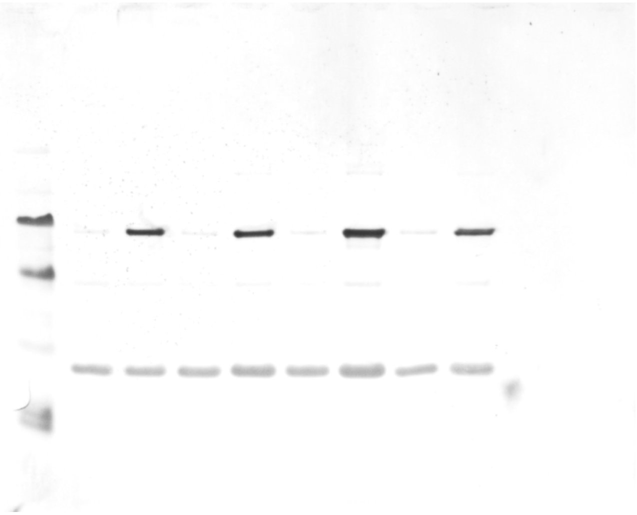

LRR3 supernatant

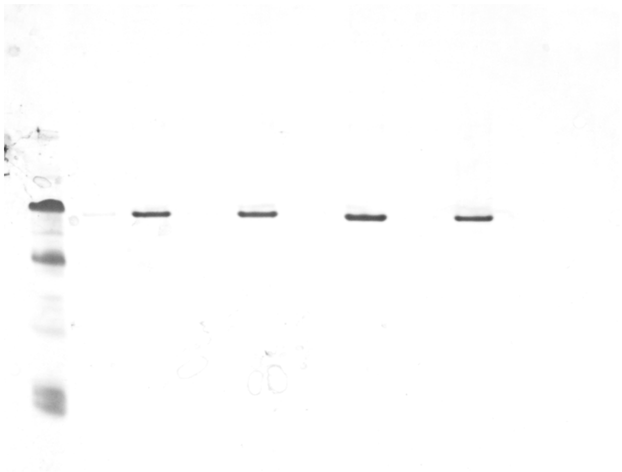

1. Yp 769 +Ca^{2+} (D28x mini pCD1)
2. Yp 769 –Ca^{2+}
3. Yp 769 + pflag-CTC +Ca^{2+}
4. Yp 769 + pflag-CTC -$Ca^{2\ +}$
5. Yp 769 + pYlrC-flag-CTC +Ca^{2+}
6. Yp 769 + pYlrC-flag-CTC -Ca^{2+}
7. Yp 769 + pYlrCΔSS-flag-CTC +Ca^{2+}
8. Yp 769 + pYlrCΔSS-flag-CTC +Ca^{2}

1 mM IPTG
α-LcrV monoclonal

whole cell

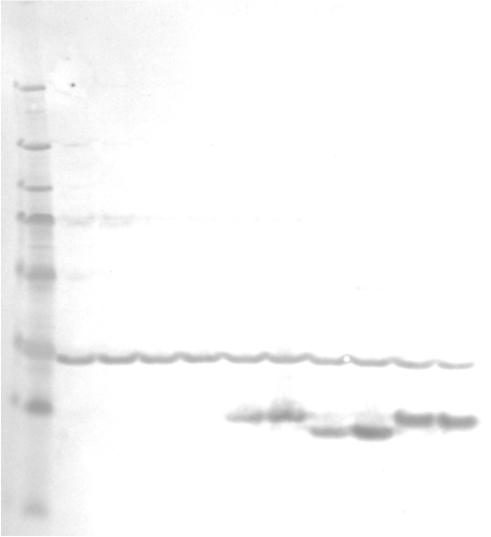

1 2 3 4 5 6 7 8 9 10

supernatant

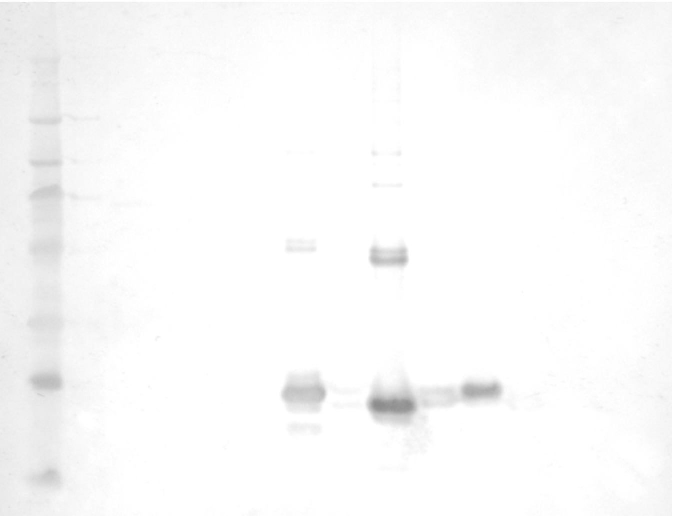

1 2 3 4 5 6 7 8 9 10

1. Yp 769 +Ca^{2+} (D28x mini pCD1)
2. Yp 769 –Ca^{2+}
3. Yp 769 + pYopE-ELK+Ca^{2+}
4. Yp 769 + pYopE-ELK -Ca^{2+}
5. Yp 769 + pLRR1-ELK +Ca^{2+}
6. Yp 769 + pLRR1-ELK -Ca^{2+}
7. Yp 769 + pLRR2-ELK +Ca^{2+}
8. Yp 769 + pLRR2-ELK -Ca^{2+}
9. Yp 769 + pLRR3-ELK +Ca^{2+}
10. Yp 769 + pLRR3-ELK -Ca^{2+}

20% arabinose
α-Elk monoclonal
